# Experiences of core outcome set developers on including stakeholders from low- and middle-income countries: An online survey

**DOI:** 10.1371/journal.pgph.0003365

**Published:** 2024-06-20

**Authors:** Jamlick Karumbi, Sarah Gorst, David Gathara, Bridget Young, Paula Williamson

**Affiliations:** 1 Department of Health Data Science, University of Liverpool, Liverpool, United Kingdom; 2 Health Systems Research, KEMRI-Wellcome Trust Research Programme, Nairobi, Kenya; 3 Centre for Maternal, Adolescent, Reproductive & Child Health (MARCH), London School of Hygiene and Tropical Medicine, London, United Kingdom; 4 Department of Public Health, Policy and Systems, University of Liverpool, Liverpool, United Kingdom; University of Bristol, UNITED KINGDOM

## Abstract

Core outcome set (COS) development and use enhances comparability of research findings. It may also enhance the translation of research into practice and reduce research waste. However, there is limited involvement of stakeholders from low- and middle-income countries (LMICs) in COS development and use. In this study, we explored the experiences of researchers in COS development projects who included stakeholders from LMICs. Online survey conducted in English of 70 COS developers from HICs who had included LMIC stakeholders in the process of developing a COS, published before the end of 2019. Respondents were identified from the COMET database and sent a link to the survey via a personalised email. Quantitative data were analysed using simple descriptive statistics. Qualitative data analysis was based on qualitative content analysis. There were 37 respondents yielding a 53% overall response rate. Analysis was limited to the responses related to 29 COS developed in the years 2015 to 2019, to reduce the potential for recall bias for earlier COS. Most respondents 20/29 (69%) were researchers. Determining ‘what to measure’ was reported as the most common stage of inclusion of LMIC stakeholders. Respondents cited (24/29, 83%) their ongoing collaborations with LMIC stakeholders such as clinicians or researchers as their main rationale for including LMICs stakeholders and reported that translation of the Delphi into languages other than English may be useful to enhance wider stakeholder participation. Involvement of LMIC stakeholders only in the later stages of COS development, lack of adequate resources to support their involvement, and lack of networks and contacts were thought to limit fuller participation of stakeholders from LMICs. To improve the involvement of LMIC stakeholders in COS development and use, COS developers need to raise awareness on the utility of COS. The need for and feasibility of translation into multiple languages warrants further discussion.

## Introduction

### What are COS and why are they useful?

Measurement and reporting of different outcomes in a given area introduces heterogeneity and can be subject to selective outcome reporting (where researchers publish only a subset of outcomes examined, often those with positive results, leading to outcome reporting bias) [1]. Evidence informed health care relies on collation of evidence about the efficacy and safety of interventions. In situations where there is a lot of heterogeneity in outcome measurement, the ability to combine, compare and contrast findings from different studies is limited [2]. When researchers report only a selection of available outcomes, they limit transparency, interpretability, and implementation of their research findings [3, 4].

Core outcome sets (COS) are standardized sets of outcomes that describe the minimum outcomes that should be measured and reported in research and/or clinical practice in a given area of health [5]. In situations where researchers measure and report COS, they facilitate evidence synthesis and translation, and reduce research waste [6, 7].

### COS development process

Developing a COS involves several steps as described in the COMET Handbook and there are standards to guide the process, the Core Outcome Set-STAndards for Development (COS-STAD) recommendations [8]. In summary, these steps are: (i) Determination of the scope (a description of the specific area of health or healthcare that the COS is to apply to) and need for a specific COS. (ii) Literature review of all available literature in that area to identify; existing works on the area; existing outcomes and/or outcomes that are important to patients. (iii) Based on the identified outcomes, consensus building to generate a common understanding on ‘what’, ‘how’ and ‘when’ to measure [5]. The most common consensus method is through a Delphi study followed by a consensus meeting [9–11]. The chosen method should have methodological rigor and ensure that a diverse range of opinions are considered.

### Stakeholder engagement

Ideally, COS development should include the perspectives of key stakeholders, including clinicians and patients/members of the public as well as researchers. This helps to ensure that all important and relevant outcomes are included in the COS thereby enhancing the likelihood of COS implementation across different settings. COS are more likely to be applicable within those countries that the stakeholders in the development process are from. Developers should endeavour to include stakeholders from countries where the prevalence or burden of the disease/condition for which they are developing a COS is high [12].

The Core Outcome Measures in Effectiveness Trials (COMET) Initiative brings together published and ongoing COS in a free, publicly available database (www.comet-initiative.org). Over 500 published COS studies are included in the COMET database to date.

Though there have been improvements in inclusion of low- and middle-income countries (LMICs) stakeholders in COS development, this remains suboptimal with only one in every five COS having participants from LMICs [10]. It is therefore important to develop strategies that can lead to improved development and use of COS in LMICs.

Understanding the experiences of COS developers who have engaged LMIC stakeholders in COS development provides a foundation for the development of approaches that could see an increase in COS development and use in LMICs. The aim of this study was to explore the experiences of those researchers (predominantly from high income countries since only four of 75 COS with LMIC participants had been initiated from LMICs [10]) in involving people from LMICs in the design and conduct of COS development projects.

## Methods

### Design

This was a cross-sectional online survey conducted in English and included some brief demographic questions, before enquiring about experiences of including stakeholders from LMICs in COS development. Stakeholders could be either, researchers, clinicians, or patients/members of the public and inclusion could be giving their views on any of the COS development stages: determining the scope of the COS; development of the protocol for the COS; determining what to measure or how to measure the COS. This study report is guided by the Checklist for Reporting of Survey Studies (CROSS) [13].

### Data collection methods

We settled on using a survey as it is inexpensive and allowed us to engage with many COS developers at the same time. Based on the finding that only one in every four COS developed have included LMIC stakeholders, we developed questions to gain a better understanding of the choice, rationale, and challenges in including LMIC stakeholders in COS development. The lead author (JK) designed the questionnaire with a mix of closed and open-ended questions and piloted it with other author team members. We kept the questionnaire relatively short (9 questions), and a photo of the lead author was included in the body of the questionnaire to enhance response [14]. The consenting process was ‘written’ through provision of a response (yes/no) to the opening statement of the questionnaire “I consent to participate in the survey (yes/no). Participants were required to respond by ticking (writing) a ‘yes’ box if they consented to participate in the survey. The survey terminated immediately for those who did not consent (those who ticked ‘no’).

The list of the questions can be found in **S1 Text**. We collected data between April and May 2021 using the using the JISC online surveys (https://www.onlinesurveys.ac.uk/) platform.

Additional information was obtained from the COMET Initiative database relating to date of COS development; geographical location of the respondents and the included LMIC stakeholders; whether the COS developers had considered how to implement the COS; and the intended use of the COS.

### Participant selection and recruitment

The survey was targeted at COS developers who had included LMIC stakeholders in the COS development as identified in a systematic review [10]. Seventy-five COS had included stakeholders from LMICs (as classified by the Organisation for Economic Co-operation and Development [15]. The survey was sent to corresponding authors of these COS as a link within a personalised email, inviting them to participate. A participant information sheet explaining the purpose of the survey, was included in the email invite. Where the corresponding author was not reachable (bounced email) other co-authors were contacted using any available email contact in the COMET Initiative database and/or in the COS publication. We personalised the email invites by briefly describing the COS that the developer had worked on and sent email reminders two weeks and one week before the survey closed to increase the chances of response.

### Ethics approval and consent to participate

Ethical approval was granted by the Health and Life Sciences Research Ethics Committee (Human participants, tissues, and databases) at the University of Liverpool on 19th November 2020 (reference 7661). All survey participants were provided with participant information as an attachment to the email inviting them to participate in the survey. The consenting process was ‘written’ through provision of a response (yes/no) to the opening statement of the questionnaire “I consent to participate in the survey (yes/no). Participants were required to respond by ticking (writing) a ‘yes’ box if they consented to participate in the survey. The survey terminated immediately for those who did not consent (those who ticked ‘no’).

### Analysis of survey responses

Quantitative data from the closed questions were analysed using simple descriptive statistics, including counts and percentages. Analysis of qualitative data (responses to the open-ended questions) was informed by qualitative content analysis and used deductive approaches whereby the themes were pre-determined by the questions in the survey [16]. All free text was extracted on to a word document which JK read multiple times to organize the text into some initial themes based on the questions in the survey and the author team reviewed the data and its assignment to the identified themes.

Most of the qualitative data comprised responses to two open ended questions. These were question 10 ‘What do you think can be done by COS developers to improve participation from LMICs?’ and question 13 *‘*If you were to develop another COS would you do anything differently?’

Excerpts from respondents’ free text responses are presented accompanied with their unique identification numbers. Respondents were assigned numbers 1 to 37 based on the recency of COS development; 1 being the most recent and 37 being the earliest. Free text responses are labelled as respondent number. question number. year that the COS was developed. For example, 1.1.2017 indicates response by respondent number 1 to question number 1 relating to a COS developed in 2017. To help maintain anonymity, small sections of text were omitted, and these are presented as square brackets (.…).

BY provided guidance on the analysis and reporting of qualitative data. The other authors DG, PW and SG provided their perspectives on the analysis and reporting of the data to help reduce domination of a single perspective on the analysis and discussions.

## Results

### General descriptions

The survey was sent to the developers of 75 COS. Five developers were each involved in two COS projects and were asked to respond to just one survey relating to one of their COS. Therefore, a maximum of 70 responses were expected.

Of these, there were 37 responses yielding a 53% overall response rate. The response rate was lower for COS developed pre-2015 (8/30; 27%) compared to 2015–2019 (29/40; 73%). We limited the analysis to the responses on the 29 COS that were developed in the years 2015 to 2019, to reduce the potential for recall bias for earlier COS. Table 1 provides a description of the 29 survey respondents.; the 8 responses related to pre-2015 COS will be described narratively. All 37 respondents provided at least one free text response and collectively responses comprised a total of 3,808 words. All free text responses are presented in **S2 Text**.

**Table 1:** Respondent characteristics.

Description		N = 29 (%)
Main profession	Health Care Practitioner	9 (31)
	Researcher/Academic	20 (69)
Time spent in the main profession	median [IQR]	13 [9–20] years
Country of respondent	Australia	5 (17)
	Belgium	1 (3)
	Denmark	1 (3)
	Germany	2 (7)
	Ireland	1 (3)
	Netherlands	4 (14)
	Poland	1 (3)
	Sri Lanka	1 (3)
	Sweden	1 (3)
	UK	8 (29)
	USA	4 (14)
Region of LMIC stakeholders included in the COS	South America	17 (59)^$^
	Asia	22 (76)
	Africa	17 (59)

^$^ The same COS study could have participants from all the three location categories as such the denominator for each category is 29 (respondents from 2015–2019) and does not add up to 100%.

Table 2 provides a summary of the responses to the survey questions related to COS development. Detailed description of this responses are provided per survey question.

**Table 2:** Survey responses to COS questions.

Survey question	Responses description	N = 29^#^ (%)
Choice of disease condition	Burden of disease in my country	7 (24)
	Burden of disease globally	16 (55)
	Personal clinical/research interest in the condition	23 (79)
	Grant/Funder requirement	1 (3)
	Other	2 (5)^£^
Rationale for including LMIC stakeholders	Prevalence of the disease/condition in those LMICs	18 (62)
	Working collaborations with LMIC stakeholders such as clinicians or researchers	24 (83)
	Suggestion by high income country (HIC) colleagues	4 (14)
	Requirement by the funding agency	1 (3)
	Other	11 (30)^£^
Stage of COS development	Determining the scope of the COS, i.e., as part of the research team	8 (28)
	Development of the protocol for the COS–as part of the research team	7 (24)
	Determining ‘what to measure’, i.e., giving their views on what to measure	29 (100)
	Determining ‘how to measure’ the COS, i.e., giving their views on how to measure	15 (51)
Translation of the Delphi to other non-English languages	Yes	9 (31)^£^
Reasons for non-translation (n = 20)	All participants were English speaking	11 (55)
	Time constraints	9 (45)
	Financial constraints	8 (40)
	Other	3 (15)^£^
LMIC stakeholders struggled to participate	Yes	8 (28)^£^
Consideration of COS implementation	Yes	15 (52)^£^

^**#**^ The denominator was 29, however where questions allowed multiple answers, the row totals and percentages for each category will be more than 29 and 100% respectively.

^£^ See further details of the responses in S2 Text.

### Choice of disease condition

**Fig 1:**
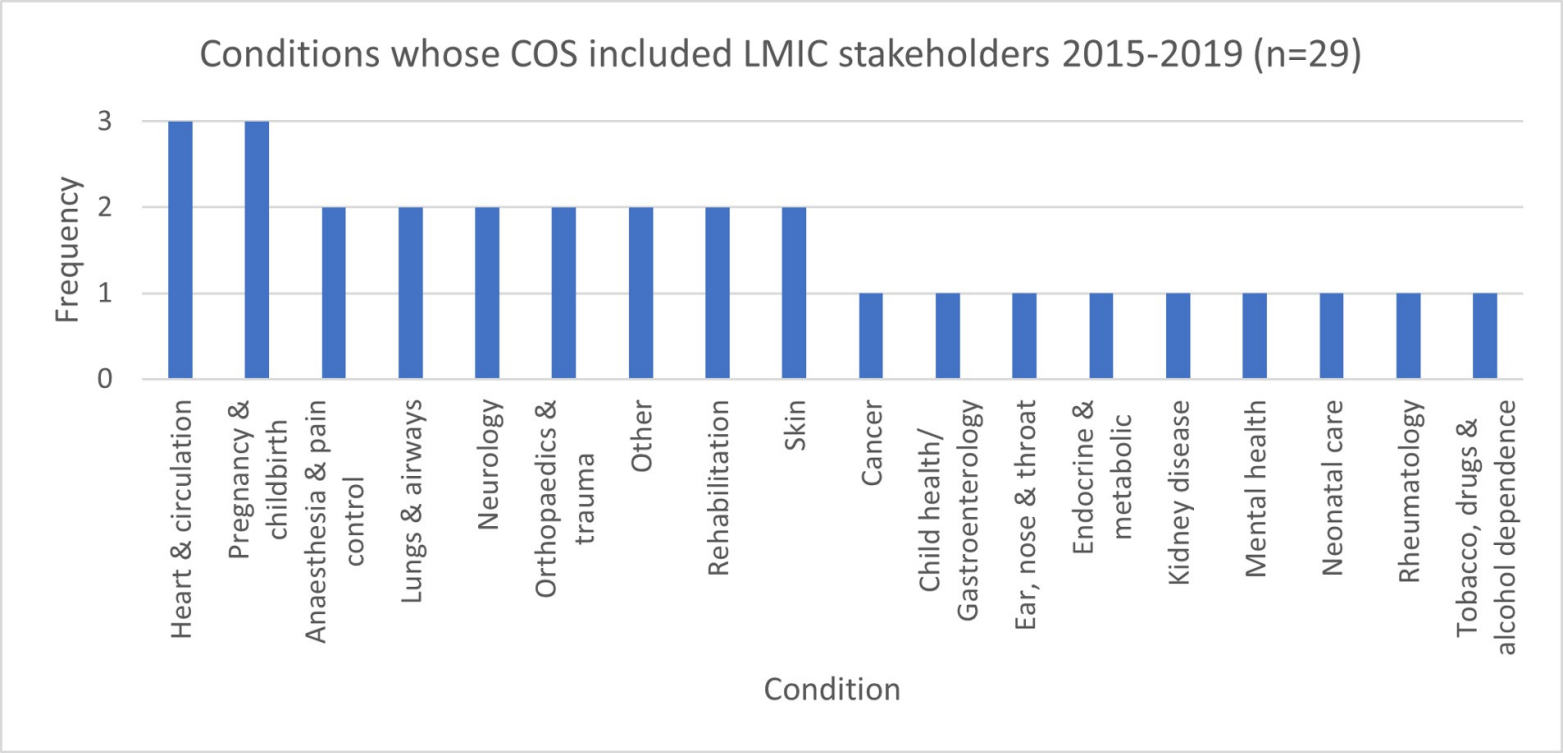
Conditions whose COS (published 2015–2019) included stakeholders from LMICs.

Fig 1 shows the conditions for which a COS was developed where stakeholders from LMICs were included. Respondents provided a range of reasons as to why a given disease or condition was chosen for COS development. Most (23/29; 79%) reported a personal clinical or research interest in the area; global burden of disease was also a reason for the majority.

### Rationale for including LMIC stakeholders

Working collaborations with LMIC stakeholders such as clinicians or researchers (24/29; 82%), and prevalence of the condition in the LMICs (18/29; 62%) were most often cited by respondents as reasons for including LMIC participants in the development of the COS. The need for a COS that is useful for everyone/global use was also a fairly common reason for having the LMIC stakeholders in the COS development process (8/29 respondents).

“The COS should be universally applicable not only in HIC’” (2.7.2019)

“It’s just the right thing to do if you want to create a product that’s useful to everyone.” (19.7.2017)

### Stage of COS development

All COS developers reported including the LMIC stakeholders as participants in determining what to measure (29/29; 100%). A smaller number (7/29; 24%) involved LMIC stakeholders as members of the research team in determining the scope and designing the protocol for the development of the COS.

### Translation of the Delphi into languages other than English

Nine respondents indicated that they considered translating the Delphi into non-English languages when they were developing the COS, of whom six reported that they did translate the Delphi. Of these nine, most reported that they had included patients as participants in their COS and the translation enabled wider participation. Of all respondents over half reported that they did not translate the Delphi as they believed all the participants were comfortable with English language.

‘English is usually ok for researchers as most research is reported in English language journals. However, for clinicians and people with the lived experience (especially those with the added difficulties of (….)) translations really are necessary. This is something that we are trying to do more often as our work progresses.’ (25.11.2016)

Respondents indicated that the translation had also helped with local dissemination of the COS and facilitated wider participation and implementation of the COS. However, they also noted cost and time constraints on translation—almost half of the respondents who did not translate cited financial and cost implications as the main challenges.

‘I have discussed generally with colleagues the barriers to implementation through the lack of translated resources in the language of the LMICs that might want to use them—especially in populous countries such as (.…) and (.…).’ (10.12.2018)

### Challenges of involving LMIC stakeholders and suggested solutions

#### Lack of contacts in LMICs

When asked about the challenges of involving LMIC stakeholders, respondents pointed to a lack of available networks and groups to work with for the specific disease/condition.

‘No one formally declined to take part, however there were certainly barriers to participation for some. It was often difficult to "link in" with appropriate networks or groups in countries where (.…) services and (.…) are still developing (or do not currently exist). Language was also a barrier.’ (25.8.2016)

Some respondents suggested that developing and using personal relationships to build trust with stakeholders in LMICs, use of professional organization/networks, and engagement of patient groups could help improve inclusion of LMIC stakeholders in the COS discourse.

They also suggested that inclusion of LMIC stakeholders early in the process, for example in determination of the scope, and identification of champions for COS within the LMICs, could help strengthen the already existing collaborations.

‘‘It is best to develop personal relationships with researchers in LMIC and then work with them to achieve what they need.’ (23.10.2016)

“get LMIC members involved early on in the COS development process (in general they are keen to be involved in international collaborations)—there is a need for the LMIC participants to see the benefits to be involved e.g. ensuring co-authorship upfront in the development stage—it is also important to get key respected figure in LMIC involved, such that they have contacts/ influence and are able to get e.g. patient advocacy groups involved as well” (18.10.2017)

One respondent felt that mortality of patient participants could have led to a declined response in the follow-up Delphi.

#### Lack of funding

A lack of financial resources was noted by respondents as limiting the participation by LMIC stakeholders when face-to-face meetings were held, especially during conferences.

‘Our research was conducted during a world congress. Sadly, colleagues from LMICs are often not able to travel to the congress due to costs (registration, accommodation, travel expenses). The Congress has recognised inequalities in people attending the conference and acted upon it.’ (3.10.2019)

Respondents suggested that one solution would be to make greater use of electronic methodologies (for example emails, virtual platforms like Zoom, Google meets etc) for consensus building.

#### Sensitization on the utility of COS

Some respondents commented about how sensitization or ‘education’ on the importance of, and methodologies for developing COS could enhance buy in from LMIC stakeholders.

‘education about importance of COS, what COS is, methodology’ (11.10.2018)

### Provision of support to enable wider stakeholder inclusion

Respondents expressed the need for guidance on best practice for enhancing inclusion of LMIC stakeholders in COS development.

‘Access to countries other than those of the investigators is a challenge even where patient or professional organisations exist. Guidance on how best to engage with existing organisations could be helpful but perhaps more so focusing on including stakeholders from relevant countries in a broader steering committee who can engage with the project and contribute to its designs and delivery from the pre-protocol stage.’ (8.10.2019)

One respondent noted that capacity building in research more widely, including how to communicate to lay public and patients, was something that had helped in their COS development process.

‘One strategy that we have used is developing a "tool kit" to support partners in other countries to apply for ethics, and run research processes locally, which then contribute to the bigger COS development process. This is also a great way of helping to build research capacity in these locations (….) Again, this is where we have developed tool kits so that we can run groups face-to-face in international locations. ‘ (25.10.2016)

### Implementation of COS

Fifteen respondents indicated that they had considered issues related to implementation of the COS in participating LMIC settings. Most reported that measurement of the core outcomes and data collection in the LMIC setting might be a challenge, due either to the need for translation and cultural adaptation of the measurement instruments, the requirement for high-tech machinery to measure some outcomes or the need to have paper versions for data collection in areas without internet connectivity. However, they commented that, in places where there are existing networks and collaborations, sensitization on COS utility might improve implementation processes.

‘…Dissemination strategies to encourage utilisation of the COS. This included using our existing networks/ collaborators to promote the COS in each country. Access to COS—providing a choice of paper version or electronic versions according to the country’s local preference and access to technology. How to promote engagement and buy in’ (16.12.2017)

## Discussion

This is the first study specifically exploring the experiences of COS developers who have included LMIC stakeholders in the process of COS development. It is part of a wider project to inform the development and use of COS in LMICs.

The choice of disease/conditions for which COS were developed is still largely driven by personal clinical or research interest. It is possible that a COS developer working on a given COS may already have contacts in LMICs (due to a common research interest) and was therefore able to include them in the COS development process. This resonates with findings from a review of evidence on inclusion of LMIC stakeholders in COS development, where personal clinical or research interests was the main driver for the COS that was developed [10]. Even though a funder’s requirement for using a COS was not widely cited in the survey by respondents, the situation may change as more and more funders and journals endorse the use of COS [17, 18].

Findings from this survey, indicate that even though LMIC stakeholders were part of the COS development, they are mainly included at the determination of what to measure, i.e., during the consensus building process of what outcomes will be in the COS; rarely are they involved during scope determination or the protocol development process. Indeed, one respondent suggested that improved use of COS in LMICs would depend on having LMIC stakeholders included from the stage when the scope of a given COS is determined. This is similar to what was reported in the analysis of data for the COS for burns [19], demonstrating the need for involvement of LMIC stakeholders. Davies et al. posit that even though there is no consensus whether separate COS should be developed for HICs and LMICs, when a decision is made to have a globally acceptable and usable COS, then it is essential to ensure complete collaboration with stakeholders across HIC and LMICs. This collaboration should span all aspects from protocol development, managing projects, and participating in projects, such as the Delphi survey and consensus meeting [20]. We suggest that to enhance buy in and enhance this collaboration, having individuals who can champion the inclusion of LMIC stakeholders in COS development and use, can lead to more inclusion of LMIC stakeholders in COS development and use process. Such individuals could help to build collaborations or engage relevant networks as the COS project is being initiated may also provide instrumental advice on the need for adaptation of a given COS in a particular setting.

Most COS developers did not translate the Delphi into languages other than English language. They believed their participants were comfortable with English language as almost all were clinicians and researchers. Survey responses indicated some of the main drivers of the COS development were personal clinical and researcher interest and working collaborations with researchers and clinicians in the LMICs. One interpretation is that to include stakeholders from LMICs, COS developers largely limited inclusion to colleagues from LMICs who are conversant with English language with patient and public participants being mainly drawn from HICs. This introduces inequity since the views of public participants from LMICs may differ from those of public participants in HICs. With the move to have more patient and public participation in COS development, it is important to consider ways of ensuring Delphi surveys are translated into locally understandable languages to facilitate more equitable patient engagement as highlighted by other research works [21, 22].

There were a few developers who struggled to include stakeholders from LMICs in COS development. The challenges they cited included:

(i) Lack of specific contacts in the target LMICs. In a majority of LMICs, there are no formal patient support groups for specific diseases/conditions and even where existent they are not very active. This may limit the inclusion of patients in COS development. This echoes the findings of a review of studies including international participants in COS development, where Lee and colleagues reported that LMICs studies were more likely to involve healthcare professionals as opposed to patients when compared to HIC studies [23]. (ii) Mortality of patient participants leading to a lack of response in a follow-up Delphi round. This is an interesting finding especially when developing COS for disease conditions that disproportionately affect LMICs or conditions that have a high fatality rate. It would be useful to see if future work, for example ongoing work for COVID-19 COS [24], report this as limitation to the response rate in Delphi studies where there are patient participants. (iii) Professional groups are often based on common conditions in a given setting. For example, if the health system of a country has several paediatric haemato-oncologists, they are likely to form a network which integrates with similar networks in other countries. In most LMICs, specialised and subspecialized cadres are under resourced, and this may hinder their wider inclusion in a COS for a specific condition [25]. However, engagement with professional networks where available has been useful in enabling participation in research and uptake of best practices by the professional in groups like the Clinical Information Network in Kenya [26]. Such networks could provide important contacts and a potential model for COS development in LMICs.

Lack of funding will hamper the inclusion of LMICs stakeholders in COS development. Funding will be required for awareness raising and training about COS generally, for translation of specific study materials into local languages, provision of access to survey software as appropriate and travel expenses for meetings if held in person. It is therefore important that when a COS developer plans to include LMIC stakeholders, there are enough resources to cater for their full participation from scope definition to the determination of ‘how to measure’ the agreed upon outcomes. One suggestion is to make better use of online platforms like emails and video conferences to ensure wider participation. Guidance on conduct of online consensus meetings for COS development has been developed [27], although consideration of internet connectivity issues is needed especially in some LMICs and of different time zones and the need to plan several meetings.

As pointed out by one of the respondents, there is need to raise awareness and enhance education on COS in LMICs. This could be informed by undertaking a survey to explore what LMIC researchers and practitioners know about COS and the gaps in their knowledge and using this information to craft strategies to introduce COS and their purpose for those who are not familiar with them. Additionally, an assessment of barriers and facilitators of using COS for those who are familiar with COS would enable the crafting of strategies to enhance the uptake of COS. Some plausible methods suggested by survey respondents include having a toolkit to help build capacity not only in general research processes (ethics application, plain language communication with lay public etc), but also having COS research development process being part of the toolkit. This would then help in integrating COS research as part of the general research training and facilitate in sessions to popularise COS either during annual professional association meetings or in continuous medical education sessions with the researchers and practitioners [28]. Based on these findings and those of LMIC stakeholders’ experiences in using COS [29], we hope to develop a model that can be used in developing COS in LMICs.

### Study limitations

We acknowledge some study limitations. There may have been recall bias for respondents. To limit the effect of recall and response bias, the analysis has been limited to the 2015–2019 COS. The sample size was small and does not allow for any major quantitative inferences. However, our sampling frame was for all COS developers who had included LMIC participants as of the end of 2019. It is possible that there are new COS developed since 2019 and the perspectives and inferences may change.

There was a disconnect between the survey reported level of inclusion in the ‘how to measure’ stage (15/29) and the data that is in the COMET database (7/29). One possible explanation is that between the time that the COS was published and when the survey was launched, some authors may have considered taking the next step of undertaking the work on the how to measure stage and therefore answered that the LMICs were involved.

Another limitation is that this survey only includes responses from COS projects where LMIC stakeholders were involved. It does not include data from other COS developers relating to why LMIC representation was not possible in their projects. Even though this may have led to missing some possible barriers of LMIC stakeholder inclusion, we conducted a follow-up survey with LMIC stakeholders on experiences of COS development and use and there were similar barriers to those reported in this work [29].

Finally, five authors had developed more than one COS and responded to only one COS survey. However, since we were seeking general experiences of inclusion of LMIC stakeholders this is unlikely to have affected the findings.

Despite these limitations, this work provides a starting point for engaging in further work to compare these findings with the experiences of LMIC stakeholders participating in COS development, with the aim of developing strategies that would improve COS development and uptake in LMICs.

## Conclusion

This survey has shown that despite the increase in inclusion of LMIC stakeholders in COS development previously noted, there are still challenges to be addressed. Involvement of LMIC stakeholders only in the later stages of COS development, lack of adequate resources to support their involvement, and lack of networks and contacts limit fuller participation within LMICs. This survey highlights the need to have further engagements with LMIC participants on the utility of COS, and the requirement for and feasibility of translation of COS development materials and the COS.

## Supporting information

S1 Text All survey questions. (PDF)

S2 Text Responses to all open-ended questions. (PDF)

S1 Checklist Inclusivity in global research. (PDF)
