# Supplementary material for: Experiences of core outcome set developers on including stakeholders from low- and middle-income countries: An online survey
Source: PLOS Glob Public Health. 2024 Jun 20;4(6):e0003365. doi: 10.1371/journal.pgph.0003365 (PMC11189180; doi:10.1371/journal.pgph.0003365)
Supplement: S1 Text — (PDF) [file pgph.0003365.s001.pdf]

Survey questions.

Exploring the experiences of core outcome set developers who have included stakeholders from low- and middle-income countries as part of the development process.

Page 1: Consenting page

Page 2: Basic information (Only, for those responding yes to the consent form)

Page 3: General characteristics

3. What is your main professional role? (By main professional role we mean the aspects of your role where most of your professional time is spent) ***(Required)***

(Required)

☐ a. Health Care Practitioner

☐ b. Researcher/Academic

☐ c. Other

i. If you selected Other, please specify: ***(Required)***

4. How long have you been involved in your main professional role? (Time in years [as an integer between 0 to 100]) ***(Required)***

Please enter a whole number (integer).

Page 4: Choice of disease/condition for the COS you developed.

5. What informed the choice of the disease/health condition for which you developed your COS? (tick all that apply) ***(Required)***

☐ a. Burden of disease in my country

☐ b. Burden of disease globally

☐ c. Personal clinical/research interest in the condition

☐ d. Grant/Funder requirement

☐ e. Other

If you selected Other, please specify: ***(Required)***

Page 5: Stakeholders from low- and middle- income countries

We would like to ask you a few questions on the LMIC stakeholders that you included in your survey.

6. What was the rationale for including stakeholders from LMICs in developing your COS? (tick all that apply) ***(Required)***

- ☐ a. Prevalence of the disease/condition in those LMICs
- ☐ b. Working collaborations with LMIC stakeholders such as clinicians or researchers
- ☐ c. Suggestion by High Income Country (HIC) colleagues
- ☐ d. Requirement by the funding agency
- ☐ e. Other

   i. If you selected Other, please specify: ***(Required)***

7. Did any invited countries or stakeholder groups decline, as a whole, or struggle to take part at all? ***(Required)***

- ○ Yes
- ○ No

   a. If yes, please provide details ***(Required)***

8. At what stages of COS development did you include stakeholders from LMICs? (tick all that apply) ***(Required)***

- ☐ a. Determining the scope of the COS, i.e., as part of the research team.
- ☐ b. Development of the protocol for the development of the COS – the 'what' to measure, i.e., as part of the research team.
- ☐ c. Determining 'what to measure', i.e., giving their views/opinion on what to measure.
- ☐ d. Determination of 'how to measure' the COS, i.e., giving their views/opinion on how to measure.
- ☐ e. Other

ii. If you selected Other, please specify: ***(Required)***

9. What do you think can be done by COS developers to improve participation from LMICs? (*Please give an idea of any challenges you faced. Please note whether your responses are in relation to the participation of particular stakeholder groups, particular countries, or particular methods that you used)* ***(Required)***

10. Did you consider translating the Delphi from English to other languages? ***(Required)***

○ Yes

○ No

i. If Yes, why? ***(Required)***

ii. If No, why? ***(Required)***

☐ a. All participants were English speaking.

☐ b. Time constraints

☐ c. Financial constraints

☐ d. Other

I. If you selected Other, please specify: ***(Required)***

11. Have you considered issues related to implementation of the COS in the participating LMICs? Required

○ Yes

○ No

a. If yes, what did you consider? ***(Required)***

12. If you were to develop another COS would you do anything differently? ***(Required)***
