# Supplementary material for: Experiences of core outcome set developers on including stakeholders from low- and middle-income countries: An online survey
Source: PLOS Glob Public Health. 2024 Jun 20;4(6):e0003365. doi: 10.1371/journal.pgph.0003365 (PMC11189180; doi:10.1371/journal.pgph.0003365)
Supplement: S2 Text — (PDF) [file pgph.0003365.s002.pdf]

Supplementary information.

S1 File **All questions and responses (verbatim)**

**Question 6. What informed the choice of the disease/health condition for which you developed your COS?**	
21.6.2017	'Design of clinical trials'
35.6.2007	'Development of a clinical service in context of a research project'
**Question 7. What was the rationale for including stakeholders from LMICs in developing your COS?**	
2.7.2019	'The COS should be universally applicable not only in HIC'
8.7.2019	'We wanted global input into the COS and contacted all organizations registered with the international [...] federation'
11.7.2018	'Global involvement'
13.7.2018	'Aimed to represent as global as possible'
15.7.2017	'Global snowball sampling'
19.7.2017	'It's just the right thing to do if you want to create a product that's useful to everyone.'
25.7.2016	'Throughout the […] COS project our sampling strategy was to include stakeholders from all world regions (as defined by the world health organization). We were not always successful in doing this - however our goal was to maximize the cultural and linguistic diversity of participants by sampling across world regions.'
27.7.2015	'Stakeholders from LMIC were not specifically approached.'
30.7.2013	'I did not explicitly recruit stakeholders from LMIC - I used mailing lists from international organizations that included members from LMIC. In the late aughts, differences among LMIC and the US were not on my radar, although they should have been.'
35.7.2007	'For COS to be useful to many'
37.7.1997	'To have a wide representation of stakeholders.'
**Question 8. Did any invited countries or stakeholder groups decline, as a whole, or struggle to take part at all?**	
1.8.2019	'I got no response from some organizational leads when trying to recruit members for the Delphi study.'
6.8.2019	'We recruited lower than hoped for individuals in our Delphi and are in the middle of validating the cos in lmics'
8.8.2019	'Access to countries was via […] federation membership. There were many non-responders to the request to be involved in distributing the COS.'
10.8.2018	'We did not explicitly track this information during the study.'
13.8.2018	'Not all who were invited replied'
16.8.2017	'I answered yes as there is not an 'unsure' option. I wouldn't describe it as 'struggle' as participation was open to all stakeholders (global clinical and research community), and we had no information on those who didn't approach us. Attendance of meetings was limited by geographical location and availability of funding to attend. Opportunities were provided to contribute via email.'
20.8.2017	'Lower response and retention rates in non-English language surveys. Reasons are unknown. However, one of our colleagues advised that due

	to higher mortality rates, some participants did not complete the second round as they had passed away.’
25.8.2016	‘No one formally declined to take part, however there were certainly barriers to participation for some. It was often difficult to "link in" with appropriate networks or groups in countries where […] services and […] rehabilitation are still developing (or do not currently exist). Language was also a barrier.’
30.8.2013	‘Representation from LMIC was much smaller than from the US and most of Europe.’
33.8.2011	‘Could not include stakeholders from Africa.’
35.8.2007	‘I’m not sure being honest. It’s some time ago and I no longer have these records’
**Question 9. At what stages of COS development did you include stakeholders from LMICs?**	
25.9.2016	‘We are in the final stages of publishing a study which examines barriers and facilitators to COS set use - using the Theoretical domains framework. This has included a small number of participants from LMICs.’
27.9.2015	‘Stakeholders from LMIC were not specifically approached.’
30.9.2013	‘Minimal’
**What do you think can be done by COS developers to improve participation from LMICs?**	
1.10.2019	‘Reaching out to relevant stakeholders from professional organisations/societies was difficult because it required contacting the lead and asking them to disseminate the invitation. This was often met with no response. It was easier if the research team had contacts within their networks and they could reach out on a personal level. Difficulties in reaching out to […] survivors from LMIC was therefore also very difficult because it relied upon professional organisations/societies agreeing to take part and helping to recruit […] survivors.’
2.10.2019	‘I found it difficult as I didn’t find any points of contact. I’ve emailed LMIC national organizations asking them to disperse the Delphi email however that didn't happen. The response mainly came from colleagues/ acquaintances. Perhaps there is also resistance towards unknown emails (afraid of spam) or a reluctance to do online surveys. The language might also be a barrier, especially when targeting LMIC patient involvement.’
3.10.2019	‘Our Research was conducted during a world congress. Sadly, colleagues from LMICs are often not able to travel to the congress due to costs (registration, accommodation, travel expenses). The Congress has recognised inequalities in people attending the conference and acted upon it.’
4.10.2019	‘Improving network and collaboration through direct or indirect contact’
5.10.2019	‘For me it was not the inclusion of LMICs that where the focus, rather the inclusion of as many countries as possible from around the World. thank you for bringing this question to my attention.’
6.10.2019	‘Use Scielo in the review. Go to conferences ideally in LMIC countries and recruit through talking to individuals who have expertise. If you

	become a trusted individual, you will be able to successfully increase your numbers.'
7.10.2019	'Use of online platforms facilitates global participation on a large scale. Particular challenges: -Burden of disease and available treatment options can be very different in LMIC settings: priorities may be very different -LMIC settings are heterogeneous, and some healthcare settings may resemble those found in high-income settings more than other geographically local settings. -For life-long conditions (like the effects of […] care) the impact of societal setting becomes so enormous that comparison even within LMIC settings becomes difficult'
8.10.2019	'Access to countries other than those of the investigators is a challenge even where patient or professional organisations exist. Guidance on how best to engage with existing organisations could be helpful but perhaps more so focusing on including stakeholders from relevant countries in a broader steering committee who can engage with the project and contribute to its designs and delivery from the pre-protocol stage.'
9.10.2018	'I particularly struggled in finding contacts in LMIC. I believe it would be very important to include LMIC but the lack of contacts and whom to approach made it difficult.'
10.10.2018	'Identifying potential participants was a challenge as people in LMICs often do not engage with the community in developing countries, and are not active in publishing and at conferences. Therefore, they are 'invisible'. We tried to be purposeful as far as possible: We created a list of potential participants with relevant expertise using manual searches of the following information sources: first and last authors of relevant conference proceedings in last 3 years (e.g. […] Research Initiative, International [...] Seminar, British […] Association conference); corresponding authors for each clinical trial of […] identified in our previous systematic review of clinical trials for [...] interventions [...] et al., 2016; and all authors of systematic reviews of [...] (Cochrane or otherwise) published in the preceding 5 years. I have some personal contacts in Country [...] and [...] and I reached out to them asking to distribute invitations to their colleagues.'
11.10.2018	'education about importance of COS, what COS is, methodology'
12.10.2018	'In our study, the main stakeholders were the patients and their parents, and the clinicians and academics. Challenges: with patients and caregivers - explaining what is COS and the purpose of the study. Once they understood, there wasn't any problems. In LMICs patient records are not computerized. So, recruiting patients for the study has to be done physically by being present in the clinics. Telephone follow-up was done to complete the 2nd round of Delphi - this was challenging to get the parents to return our questionnaire. several follow-up calls were made. stamped envelopes with pre-printed return address were sent along with the questionnaire to increase response. Clinicians and academics - regular reminders were sent by telephone calls and emails to get the responses.'
13.10.2018	'Use all network available'

14.10.2018	'The involvement of representatives of LMICs in the COS steering group is of utmost importance. These representatives can guide the project group in its strategies for engaging LMICs stakeholders at all stages of COS design and suggest appropriate methods for interaction.'
16.10.2017	'At the time of developing this COS we did not specifically target LMIC's. Opportunity to contribute to the development of the COS was extended to the global clinical and research community for this specific condition, wherever they were based. To improve participation, we provided the opportunity to contribute electronically, for example via email or virtual communication platforms; we promoted opportunities to contribute via conferences and though professional networks; To improve participation, developers could target expertise within the LMIC via journal publications, professional networks and via professional groups within the country'
17.10.2017	'- Better use existing collaborations for inviting potential participants. - Using international conferences (such as [...] conference or [...] conference) for advertising - directly contact researchers from LMICs who might be a good fit for the working group depending on their previous research work. - offering Delphi meetings in other languages than English, for example by providing translators.'
18.10.2017	'- get LMIC members involved early on in the COS development process (in general they are keen to be involved in international collaborations) - there is a need for the LMIC participants to see the benefits to be involved e.g. ensuring co-authorship upfront in the development stage - it is also important to get key respected figure in LMIC involved, such that they have contacts/ influence and are able to get e.g. patient advocacy groups involved as well'
19.10.2017	'Keep surveys short and simple. Provide lots of training on exactly what a COS is and why it matters.'
20.10.2017	'Further consultation as to how to best engage patients/caregivers from LMICs (we conducted online surveys). Studies are needed to examine challenges/barriers.'
21.10.2017	'These COS was related to the design of clinical trials for [...] patients. So it was different from other COS for a specific condition. Specifying the outcomes was only part of our recommendations, e.g. trial design, patient inclusion/exclusion criteria, etc.'
22.10.2016	'- build networks with experts globally'
23.10.2016	'It is best to develop personal relationships with researchers in LMIC and then work with them to achieve what they need.'
24.10.2016	'Simply to think through the implementation and that LMIC should always be involved. Most conditions have no borders.'
25.10.2016	'I think the main challenge is connecting with the right people in LMICs to involve them in COS development. In our field this has been particularly difficult in Africa, South America and Asia as our collaborations and networks are not as strong in these locations. For us, incorporating the [...] COS work within the Collaboration of [...] Trialists (an international network of [...] researchers that spans more than 40 countries and 38 different languages) has helped with this. Some researchers (often in more developed countries) have voiced concerns

	that COS use in LMICs places too great a burden on researchers, however, when talking to researchers in LMICs we have often found that they want information and guidance on which outcome measures to translate/adapt - so that their resources can be more carefully used. Translation and cultural adaptation of materials is very important to facilitate participation - this is also where strong international networks are of use. One strategy that we have used is developing a "tool kit" to support partners in other countries to apply for ethics, and run research processes locally, which then contribute to the bigger COS development process. This is also a great way of helping to build research capacity in these locations. [...] is an impairment of [...] and so this has added additional challenges in the recruitment of people with the lived experience. Online surveys can present a barrier to participation due to the requirements for reading and writing, which can be impaired in people with [...]. Again, this is where we have developed tool kits so that we can run groups face-to-face in international locations. The face-to-face format makes it easier to support communication.'
26.10.2015	'- Reach clinicians and researchers from LMICs at the design stage of a COS (i.e. before defining a COS) - Find a contact point in LMICs that will be able to help you recruit other clinicians, researchers and patients to be involved during various steps of the COS development process (e.g. to participate in the Delphis)'
27.10.2015	'To identify and specifically invite the participants from LMICs.'
28.10.2015	'Clear description of the aim of the project with simple language and terminology. COS are not standard yet in HIC, but even less in LMIC. The process can be difficult for any stakeholder group, patients and clinicians especially. I had the help of language specialists. Actively approach research and healthcare communities of LMIC with interest in increasing quality of research Keep the Delphi process simple. Try to minimize duplicate/overlap in outcomes and give examples for each outcome domain. Sometimes pictures can help. Use a dephi program that work on different devices (including mobile phone) for countries with bad internet connection.'
29.10.2015	'Active recruitment of young clinicians/investigators from LMIC'
30.10.2013	'As COS guideline development continues, there should be a defined approach that encourages inclusion of LMIC stakeholders.'
31.10.2012	'Liaise with patients support groups'
32.10.2011	'Raise the consciousness of this issue. Despite being in the file of outcome measures, i have not heard this as an important issue. I have heard about need for geographic variety but not for income reasons.'
33.10.2011	'Explain relevance of global cos for their work or setting'
34.10.2008	'A disease is a disease everywhere. So, I think that in any case COS must be the same everywhere and any effort must be done to implement them in LMICs. It is a political approach, not a scientific one. Every person must receive the best treatment to reach the same target.'
35.10.2007	'Translation of documents Online engagement opportunities Authorship opportunities for colleagues'
36.10.2001	'Low-income countries have been included in more recent years of our international [...] group's 20 year history. It has taken awhile for the

	group's work to be of interest to these countries and for them to get involved. Electronic communications and meetings (like Zoom, and the pandemic) have enhanced our interactions with these countries.'
37.10.1997	'Think about it. Know researchers in the topic in LMICs.'
**Did you consider translating the Delphi from English to other languages? If yes, why?**	
5.11.2019	Yes 'we had countries included that was speaking other languages such as [...], [...] and [...].'
8.11.2019	Yes 'It was requested by one organisation. However only 1 person completed round 1 in an alternative language'
12.11.2018	Yes 'Local language was used to communicate with the patients and their parents. Clinicians and academics - used the English version without any problem'
20.11.2017	Yes 'So participants could complete the survey (they could not do it in English).'
24.11.2016	Yes 'For dissemination'
25.11.2016	Yes 'English is usually ok for researchers as most research is reported in English language journals. However for clinicians and people with the lived experience (especially those with the added difficulties of [...]) translations really are necessary. This is something that we are trying to do more often as our work progresses.'
27.11.2015	Yes 'Not everyone speaks English.'
28.11.2015	Yes 'To increase the representation. On the end this is really a lot of work. But with adequate funding I would certainly have done it' - 2015
29.11.2015	Yes 'I think [our organization] did - 2015
32.11.2011	Yes 'Our COS did not include a Delphi but other similar projects do and we have considered translations, especially for patients.'
33.11.2011	Yes 'To allow broad participation'
34.11.2008	Yes 'To spread information locally.'
**Have you considered issues related to implementation of the COS in the participating LMICs?**	
2.12.2019	'Yes, we did. Costs and accessibility to tests/diagnosis'
6.12.2019	'Cost of questionnaires was the main one. Time burden Translations and validation work outside of high income and /or English speaking countries Differing nature of how the intervention is used in the country'
7.12.2019	'-Applicability of outcome in LMIC settings -Difficulties performing outcome assessments in LMIC settings -Need for separate COS for LMIC settings'
10.12.2018	'I have discussed generally with colleagues the barriers to implementation through the lack of translated resources in the language of the LMICs that might want to use them - especially in populous countries such as [...] and [...].'
12.12.2018	'1) quality of life (QOL) was one of the COS identified in our study. We considered the feasibility of measuring QOL in the local context - this finding directed us to develop a culturally acceptable QOL tool for the local context, which was lacking at that time. 2) certain outcomes preferred by the patient/parent are unrealistic (e.g. [...]) at times.

	However, we felt that while COS are developed, patient preference should be given more importance that just developing a set of outcomes that are convenient for the clinicians/researchers but not useful/meaningless for the patients.'
13.12.208	'We have not worked much on implementation yet'
16.12.2017	'Availability of outcome measures in local languages and potentially the need to translate the COS Dissemination strategies to encourage utilisation of the COS. This included using our existing networks/ collaborators to promote the COS in each country Access to COS - providing a choice of paper version or electronic versions according to the countries local preference and access to technology How to promote engagement and buy in'
18.12.2017	'- it was discussed multiple times throughout the COS development process as to whether the COS data collection is feasible in LMIC - there is a need to balance the number of items collected and whether it's at all feasible to be collected in LMIC. - to the best of my knowledge, I do not think the COS is being routinely collected in LMIC involved in the COS development at the moment.'
19.12.2017	'We encouraged participants to think about whether measurement instruments requiring internet connectivity or high-tech machinery would be feasible in LMICs. For each survey-based instrument considered we also listed whether it was available in languages other than English.'
20.12.2017	'Cost/resources Importance/relevance'
23.12.2016	'The need to make it acceptable to users.'
25.12.2017	'See response above. There is a perception that COS use will add a burden to researchers in LMIC. However, we have often found that researchers in LMICs want guidance on how best to use their resources if they are for example considering translating/adapting a measure of the quality of life. They don't want to choose the wrong outcome measure to translate if they have finite resources. On the other hand, in some LMIC [...] research does not exist or is only just emerging and so COS use and treatment trials seem premature. We are currently studying barriers and facilitators to [...] COS use - lack of availability of translations and cultural adaptations of recommended measures is another issue.'
28.12.2015	'It was important to distinguish the 'feasibility' of the outcome collection and the 'importance' of the outcome collection. It is crucial to make sure the delphi participants understand that phase 1 'the what to measure' should NOT capture feasibility issues. Those issues are even more relevant for LMIC'
29.12.2015	'[...] organization has spent considerable efforts in implementation'
35.12.2007	'This COS was pre COMET and needs to be revised'
**If you were to develop another COS would you do anything differently?**	
Q95	'Ideally I'd like there to have been even better LMIC representation on the Delphi panel.'
1.13.2019	'Invite someone from a LMIC onto the research team'

2.13.2019	'More specific question - the systematic review component was very long More LMIC involvement - I have significantly more experience now in targeting an audience More LMIC patient involvement'
3.13.2019	'The nominal group technique asks for people attending. However, not everyone is able to attend and I would now send out a survey to participants, who were not able to attend and to groups who are underrepresented.'
4.13.2019	'No; this worked perfectly well'
5.13.2019	'I Believe that we have had a good plan in the research process. We have included patients all the way from the beginning and through the whole process which is very important. In the Delphi we included different stakeholders. Which is important both for their opinion but also when it should be implemented. Don't know what we could have done differently.'
6.13.2019	'Absolutely yes. More on the steering group and throughout every element. Co-authors on every paper arising, review, e-Delphi, final cos, costing in travel to LMIC because I honestly feel like personal meeting is the key to the door. Seek funding specifically for this engagement'
7.13.2019	'-Retention of Delphi participants is always difficult, especially when participation is only online. Keeping survey windows short, and thus reducing the overall project duration would probably have helped retention.'
8.13.2019	'I would think more about the steering committee membership and how they can help to deliver the cos study.'
9.13.2018	'Would include more LMIC'
10.13.2018	'Unsure. I'd like to involve a steering group member from somewhere like Country [...] which is a developing country and which is rapidly expanding its research activity and clinical trials work. However, the internet restrictions make communication difficult (no access to Microsoft Teams, Facebook, Zoom or WhatsApp). And the time difference is also a consideration.'
11.13.2018	'Consider issues above mention in more depth'
12.13.2018	'1) increase the sample size 2) Telephone follow-up with parents for 2nd round - I would rather prefer to wait until their next clinic visit to get their response when they come to the clinic. This will increase their response rate.'
13.13.2018	'Spend more time to get an even broader representation'
14.13.2018	'Patient and LMIC stakeholder engagement are certainly key areas for additional attention/consideration.'
17.13.2017	'Yes, I would try to include even more participants from LMICs.'
18.13.2017	'- nothing major that will be done differently - needs to ensure there is a credible group of stakeholders involved - ensure LMIC stakeholders' involvement early on if we want the COS to be implemented in LMIC'
19.13.2017	'Ideally, I'd like there to have been even better LMIC representation on the Delphi panel.'
20.13.2017	'More initial work to determine other approaches (or how to improve the process) for engagement.'

21.13.2017	'More careful crafting of the Delphi questions to better capture the groups opinions.'
22.13.2016	'Taking into account the overall goal of the project, developing a core outcome set for the use globally, I would have put effort in the collaboration with and contribution of experts from LMICs'
24.13.2016	'Wider stakeholder involvement'
25.13.2016	'I would link with a network like the Collaboration of [...] Trialists earlier and try to broaden the reach of the consensus processes by making research accessible to people who speak languages other than English.'
26.13.2015	'YES, I would seek for a more prominent involvement of patients from any country, and of more participants (not only patients) from LMICs. The number of patients in our Delphis was limited, and we were able to involve people mainly from one middle-income country (i.e. [...]) just because we had good connections there. We did not try to reach to people in other LMICs.'
27.13.2015	'Yes. More global representation of the stakeholders should be considered.'
29.13.2015	'Attempt to get buy-in from consumers (government, payers, etc) earlier in process'
30.13.2013	'I would take a more global approach now appreciating that Europe and the US do not make up the whole globe.'
31.13.2012	'Yes involve more patients'
32.13.2011	'Yes. Would aim to more purposefully include investigators and patients from LMICs.'
33.13.2011	'Include more patients from more countries'
35.13.2007	'Greater LMIC participation from beginning'
36.13.2001	'Need to consider more on the use of these in the clinic setting.'
